# Beekeeper attitudes towards the management of *Varroa destructor* in Australia

**DOI:** 10.1007/s13280-025-02307-5

**Published:** 2025-12-11

**Authors:** Cornelia Sattler, Andrew B. Barron, Theotime Colin

**Affiliations:** School of Natural Sciences, Macquarie University, Balaclava Rd, Macquarie Park, Sydney, NSW 2113 Australia

**Keywords:** *Apis mellifera*, Brood break, Non-chemical control methods, Queen caging, Sugar dusting

## Abstract

*Varroa destructor*, the most damaging pest of the western honey bee (*Apis mellifera*), arrived in Australia in June 2022. This parasitic mite weakens bees and spreads harmful viruses. We investigated Australian beekeepers’ trust and perceptions of biomechanical *Varroa* control methods to identify barriers to adoption. An online survey and in-person interviews were conducted with commercial, semi-commercial, and hobbyist beekeepers. Data were analysed using generalised linear models and content analysis. While many beekeepers expressed interest in non-chemical approaches, most relied on chemical treatments. Although 80% had heard of biomechanical options, few applied them due to concerns about time, practicality, and efficacy. Interviews revealed limited available information and context-specific challenges, including high reinfestation rates and co-infestation by small hive beetles. Our findings highlight the need for targeted education and support to promote sustainable *Varroa destructor* management and provide guidelines for future research aligned with commercial beekeepers’ needs.

**Supplementary Information:**

The online version contains supplementary material available at 10.1007/s13280-025-02307-5.

## Introduction

Inevitable and expected (Phillips 2020), the most damaging honey bee parasite—*Varroa destructor*—arrived in the port of Newcastle, New South Wales, Australia, in June 2022 (Australian Government 2025). *Varroa destructor* (hereafter *Varroa*) is a parasitic mite that develops in the brood cells of drones and worker bees and feeds on the haemolymph and fat bodies of honey bees (Nazzi and Le Conte 2016; Ramsey et al. 2019; Han et al. 2024). The mite is a vector for many viruses which harm honey bees (Rosenkranz et al. 2010).

Following the incursion in NSW, beekeepers faced pressing decisions regarding colony management, as Varroa spread rapidly to Victoria, the ACT, and Queensland (Chapman et al. 2023; ACT Government 2025; Agriculture Victoria 2025; Queensland Government 2025). While eradication measures were initially implemented, spillover to free-living colonies and illegal hive movements made eradication unfeasible, leading to the implementation of a national Transition to Management Plan in 2024 (Reuters 2023; NSW Government 2025).

The rapid spread of *Varroa* left little time for Australian beekeepers to obtain information, change practices and identify the best local management strategies. However, because *Varroa* management is key to the health and survival of Western honey bee colonies (vanEngelsdorp and Meixner 2010) a range of control methods have been developed in other parts of the world which are available to Australian beekeepers to mitigate damage to bee colonies. These include cultural controls such as breeding for resistance to *Varroa*, biomechanical controls such as brood interruption which breaks the lifecycle of the *Varroa*, and chemical control based on synthetic or organic miticides (Jack and Ellis 2021).

Chemical controls are the most popular option among beekeepers. These are often grouped into two categories: synthetic miticides (e.g., Amitraz, Tau-fluvalinate, Flumethrin) and organic compounds (e.g., hop, oxalic and formic acid, thymol and other botanical extracts) (Rosenkranz et al. 2010; Mitton et al. 2022). These methods lead, however, to significant issues. Frequent use may select for resistance in *Varroa* populations (Mitton et al. 2022), leave residues in hive products (Mullin et al. 2010), and have negative effects on honey bee health (Tihelka 2018). Other common issues with chemical control include limited options during honey flows due to food safety concerns and risks to the health of beekeepers (Mitton et al. 2022; O’Connell et al. 2025). These limitations highlight a need for alternatives to synthetic and organic chemical treatments.

Biomechanical control alternatives exist, but their rate of adoption is unknown globally. Drone brood removal is one effective biomechanical method to reduce mite loads. In this method drone foundation is introduced to the colony to encourage drone egg laying. *Varroa destructor* infest drone cells up to three times more than worker cells, and up to 6.95 more mites are typically found in a drone than in a worker cell (Calderone and Kuenen 2001). Once the cell is capped, the females are trapped inside. Before the drones emerge, the frame is removed, frozen and uncapped, removing mites from the colony (Calderone 2005). A similar biomechanical method consists in trapping mites on worker brood. In this method, the queen is caged and allowed to lay eggs only on a single frame. Most of the *Varroa* in the hive should eventually move onto this frame when it becomes the only one with brood cells. Before worker bees emerge, the frame is removed, frozen and reintroduced later. Similar to the drone brood trapping method, there can be more than one frame used consecutively to trap and remove mites in brood (Büchler et al. 2020). Both methods interrupt the mite’s life cycle and can drastically reduce the numbers of mites in hives (Calderone 2005; Büchler et al. 2020). Sugar dusting is another biomechanical method, although it is labour-intensive and its efficacy varies (Fakhimzadeh 2001). In this method, icing sugar is blown between brood frames using an air blower (Carroll and Brown 2024). The aim of this method is to coat bees in dusting sugar, possibly to promote grooming and dislodge phoretic mites (Fakhimzadeh 2001; Carroll and Brown 2024).

Despite their potential, these methods are rarely used as stand-alone solutions, partly because they are labour-intensive and time-consuming (Büchler et al. 2020; Carroll and Brown 2024). Instead, biomechanical methods are often combined with chemical or biological controls within integrated pest management frameworks (Jack and Ellis 2021; Underwood et al. 2023) Integrated pest management involves determining economic thresholds for treatment interventions, monitoring *Varroa* populations, implementing preventative methods, and applying treatments in a stepwise manner as needed (Jack and Ellis 2021). However, no studies have directly examined why the uptake of biomechanical control methods is limited. Understanding these barriers could help refine biomechanical control designs, reduce their drawbacks, and guide *Varroa* management towards more sustainable and widely adopted practices.

To gather insights into the current state of *Varroa* impact, management practices, and beekeepers’ expectations and requirements in terms of mite control, we surveyed and interviewed Australian beekeepers. We focused on three biomechanical control methods (drone brood removal, worker comb trapping, and sugar dusting) to explore opportunities for improving or diversifying control options that could be adopted by beekeepers. We determined (1) which *Varroa* management methods beekeepers use, (2) their perceptions of biomechanical methods, (3) their trust in existing chemical and biomechanical methods, and (4) the main barriers to the adoption of biomechanical methods.

Comparative studies of the effect of biotechnical methods on colony survival and performance are rare. A review by Brook (2025) compared different strategies to manage *Varroa* without chemical treatment. The authors identified successful strategies, such as locally breeding *Varroa* resistant bees acquired from other beekeepers, removing drone brood, interrupting brood before winter for colonies with high *Varroa* counts or replacing queens in colonies with high *Varroa* levels (Brook 2025). The review also emphasised that current *Varroa* management guidelines primarily focus on chemical treatments and little on alternative options like biomechanical methods (Brook 2025; The National Bee Unit 2025). Non-chemical strategies, as outlined by Brook (2025), offer a promising pathway to chemical-free beekeeping, emphasising the importance of starting with resistant colonies and employing biomechanical methods to manage initial *Varroa* loads. This is emphasised by a study in Cuba which showed that no chemical treatment of *Varroa* over 2 decades led to an increase in resistance by uncapping and removal behaviour of infested brood cells by honey bees (Luis et al. 2022).

## Materials and methods

### Online survey

Our survey was accessible online from the 10th of February 2025 to the 28th of May 2025 as a Microsoft Forms questionnaire. The survey contained 19 questions, including consent approvals and an open comment section at the end, and was divided into three parts. In the first part, we focused on demographic and operational information, including location, beekeeping experience, stock, and the focus of the operation (e.g. honey production, pollination services, or queen rearing). The second part of the survey focused on the sources of knowledge regarding mite control, and on their current practices in terms of *Varroa* checks and treatments. We also asked participants to quantify colony losses attributed to mites and their satisfaction levels with *Varroa* treatments from a list of several options (See Table 2). The third and last section of the survey focused on biomechanical *Varroa* treatment methods. Participants were presented with different statements about non-chemical methods which they rated according to their own perceptions and criteria, such as knowledge, time, practicality, and trust (Appendix S1). Next, participants were presented with three different protocols of biomechanical mite control: queen caging on comb (brood trapping), sugar dusting and drone brood trapping (Appendix S1). Participants were asked whether they would use the presented biomechanical control methods over chemical control if the methods were effective. To determine why participants might not use the presented methods, they were presented with different statements and asked to choose those that applied, or to fill an open text box for any other response (Appendix S1).

The last question was an open text box which provided participants the opportunity to share any other comments. All collected data were anonymous, but participants had the opportunity to volunteer their email addresses if they wished to receive information about future opportunities and communications relating to our projects.

### Interviews

Semi-structured, one-on-one interviews were conducted between the 17th of February 2025 and the 12th of May 2025 and focused on four main themes: Information sources and management of *Varroa*, impact of *Varroa* on beekeeping practices, perception towards biomechanical control methods and *Varroa* prevention. Participants were first asked to provide information about their background, location and the number of hives they possessed. We then asked how often they checked *Varroa* levels and how they obtained information about *Varroa* treatments. We also wanted to know whether participants noted any unusual absconding of bees (bee colonies leaving hives and relocating their nests) or increases in other pests (e.g. small hive beetles) that they attributed to *Varroa*. To gain additional insights about their satisfaction levels with different *Varroa* treatment options, participants were asked how they treated *Varroa* and how satisfied they were with the methods they were using. Finally, we wanted to know the perception of participants towards non-chemical methods and asked them what biomechanical methods they were aware of and whether they would consider trialling them. Additionally, we sought information about the main barriers to the adoption of biomechanical control methods and the minimum requirements that a biomechanical method should meet to be adopted. We asked participants whether they used any preventative methods against *Varroa*. At the end of each interview, we asked participants whether there was a topic we did not address, or additional information they wanted to share (Appendix S2). Interviews lasted no longer than one hour.

### Dissemination and participation

Our online survey was sent to all beekeeping groups, clubs and associations in New South Wales, through newsletters, emails and Facebook groups. Simultaneously, we released a project website with our online survey, which was shared within our beekeeper network and during beekeeping conferences. Additionally, a network of beekeepers established in a previous project was used to recruit interviewees, and we used snowball sampling as a non-probability sampling method (Leighton et al. 2021): At the end of each interview, participants were asked if they wanted to provide potential contacts for further interviews. Interviews were terminated when no new information was obtained. Both, interview and survey questions were piloted and validated by a group of beekeepers before the online survey and the interviews started.

### Statistical analysis

With the consent of participants, all interviews were audio-recorded and transcribed using the automated transcription feature in Adobe Premiere Pro. Transcripts were manually checked, and content analysis was conducted using a mix of deductive and inductive coding in NVivo. The codes followed the main topics: Information sources and management of *Varroa*, impact of *Varroa* on their beekeeping practice, perception towards biomechanical methods and prevention methods against *Varroa*. To test whether the use of control methods (biomechanical, synthetic or organic) depended on the occupation of participants (hobbyist, semi-commercial or commercial) we used generalised linear models with a binomial family using methods as the dependent variable and occupation as an independent variable. R Statistical Software, version 4.3.2. was used for all statistical analysis.

### Ethics approval

Ethics approval for the stakeholder interviews and online survey was granted by the Science & Engineering Subcommittee of the Macquarie University (Ref. 520251843661254—February 2025).

## Results

A summary of the drivers and barriers affecting the adoption of non-chemical methods, identified in the online survey and interviews, can be found in Table 1.

**Table 1:** Summary of online survey and interviews of barriers and drivers for using mechanical (non-chemical) *Varroa* control methods

Category	Drivers	Barriers
Perceived effectiveness	Desire for chemical-free alternatives	Scepticism about efficacy, especially in commercial settings Lack of proven results Perceived as unreliable
Knowledge and education	Interest in learning more Some awareness from governmental websites	Poor understanding of methods Confusion between "organic" and "mechanical" Misinformation (e.g., bees drowning in sugar)
Labour and practicality	Willingness to try under better conditions (e.g., lower reinfestation rates) lowering long-term chemical dependency	Time-consuming Labour-intensive Impractical for large-scale use
Information accessibility	Some find protocols understandable	Difficulty accessing reliable and easy-to-follow instructions
Brood removal methods	–	Dislike of brood sacrifice View of brood as a valuable resource
Reinfestation risk	–	High reinfestation from nearby hives or feral colonies Lack of trust in neighbours' management
Chemical trust and convenience	–	Chemicals seen as easier and more reliable Chemicals remain active in hive, reducing reinfestation risk
Economic impact	Some interest in preventative approaches (e.g., drift reduction)	Loss of income/productivity from brood removal Difficulties during pollination due to hive spacing needs (drift prevention)
Policy and context	Openness to change if future conditions stabilize	Existing protocols perceived as imported and unsuitable for Australian conditions
Peer influence and demonstration	Would adopt if shown working examples	Need to “see it work” before adopting

### Online survey

In total 130 beekeepers participated in our online survey. Out of the 130 participants, seven did not provide consent and were excluded. The majority of participants had their apiaries located in New South Wales, which represents around 1% of total beekeepers registered in NSW (14 377 recreational beekeepers, 721 commercial beekeepers). The majority of Australian beekeepers are located in NSW (Clarke and Le Feuvre 2024).

Out of the 123 participants, 70% were hobbyist beekeepers, followed by 18% part-time beekeepers and 12% commercial beekeepers. The majority of beekeepers surveyed produced honey (62%), provided pollination (22%) and reared queens or produced colonies (9%). Most beekeepers surveyed gathered information about *Varroa* treatments via beekeeping clubs and associations (81%) and government communications or workshops (80%). Beekeeping websites (53%), YouTube or video streaming platforms (43%) and social media like Facebook (36%) were other important information sources for *Varroa* treatment.

Almost half of the participants (44%) reported checking for *Varroa* presence and levels on a monthly basis. A further 19% checked their hives more than once a month, while 16% did so every 3 months. Five beekeepers reported not checking for *Varroa* in their hives at all.

### *Varroa* treatment

Despite a trend in higher uptake of biomechanical methods among non-commercial beekeepers, occupation did not significantly influence the likelihood of using biomechanical control methods. Hobbyist beekeepers had approximately twice the odds of using biomechanical methods compared to commercial beekeepers (OR = 1.97, 95% CI 0.49–13.27), and semi-commercial/part-time beekeepers had 2.44 times higher odds (95% CI 0.47–18.60). Thirty-three per cent of commercial, 31% of hobbyist and 50% of the part-time beekeepers indicated using Bayvarol (synthetic chemical flumethrin) and were satisfied with this product. Only a small percentage of hobbyist and part-time beekeepers indicated that they were not satisfied with some of the listed chemicals (see Table 2). Organic chemicals were the second most used method: oxalic acid was used by over 20% of commercial and part-time beekeepers, and by 15% of hobbyists. Biomechanical methods were the least used by beekeepers. However, when used, beekeepers indicated they were satisfied with the methods. Only a small percentage of hobbyists indicated they were not satisfied with the used biomechanical methods.

**Table 2:** Percentage of participants indicating satisfaction with the listed *Varroa* treatment methods. Results are separated by the occupation of participants (commercial, hobbyist and part time beekeeper)

Treatment method	Response	Commercial	Part-time	Hobbyist
		*n* = 15	*n* = 22	*n* = 86
*Chemical*				
Apistan	Did not use	93.3	90.9	95.3
	Used/not satisfied	0.0	0.0	1.2
	Used/satisfied	6.7	9.1	3.5
Apivar	Did not use	86.7	81.8	88.4
	Used/not satisfied	0.0	0.0	2.3
	Used/satisfied	13.3	18.2	9.3
Bayvarol	Did not use	66.7	40.9	65.1
	Used/not satisfied	0.0	9.1	3.5
	Used/satisfied	33.3	50.0	31.4
*Organic*				
Formic acid	Did not use	93.3	68.2	76.7
	Used/not satisfied	6.7	0.0	9.3
	Used/satisfied	0.0	31.8	14.0
Oxalic acid	Did not use	73.3	72.7	81.4
	Used/not satisfied	0.0	4.5	3.5
	Used/satisfied	26.7	22.7	15.1
Thymol	Did not use	100.0	95.5	94.2
	Used/not satisfied	0.0	4.5	3.5
	Used/satisfied	0.0	0.0	2.3
*Mechanical*				
Artificial swarming	Did not use	93.3	86.4	89.5
	Used/not satisfied	0.0	0.0	1.2
	Used/satisfied	6.7	13.6	9.3
Brood break	Did not use	100.0	95.5	94.2
	Used/not satisfied	0.0	0.0	1.2
	Used/satisfied	0.0	4.5	4.7
Queen caging	Did not use	100.0	86.4	96.5
	Used/not satisfied	0.0	0.0	2.3
	Used/satisfied	0.0	13.6	1.2
Sugar dusting	Did not use	86.7	90.9	84.9
	Used/not satisfied	0.0	0.0	3.5
	Used/satisfied	13.3	9.1	11.6

### Feedback on alternative methods

**Fig. 1:**
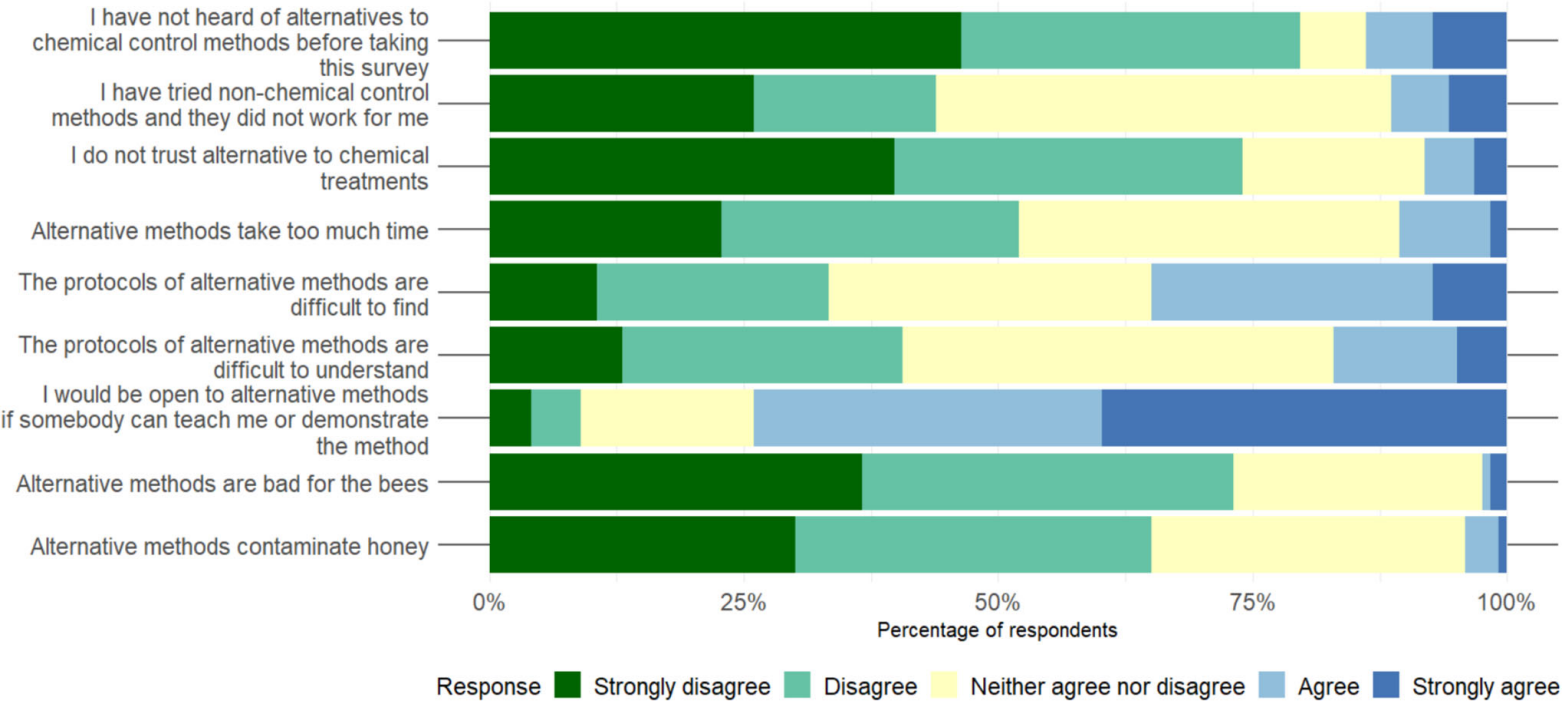
Feedback on alternative methods from survey participants

Out of 123 participants, 98 had heard of alternatives to chemical treatments prior to the survey (Fig. 1). Forty-four per cent had tried alternative methods and disagreed with the statement that these methods do not work. Overall, 74% did not agree with the statement that alternative methods cannot be trusted. Just over half (52%) disagreed that such methods are too time-consuming, 11% agreed, and 37% neither agreed nor disagreed.

When asked whether protocols on the use of alternatives to chemical treatments were easy to find and understand, 34% said they were difficult to find. Forty-one per cent considered that the protocols were not difficult to understand. For these two questions, 32% and 42% of participants, respectively, were ambivalent. Over 70% of participants were open to trying alternative methods. Only 3% and 4%, respectively, were concerned non-chemical methods could contaminate honey. Meanwhile, 24% and 31% neither agreed nor disagreed with those statements.

### Protocols of biomechanical methods

Across the three biomechanical methods, beekeepers showed similar uncertainties. Fifty-three per cent of participants indicated they would not use the comb trapping method where the queen is caged on a frame because they perceived this method as too time consuming (50% participants), impractical (24%) or were worried about the queen survival (21%). Fifty-two per cent of participants did not want to use icing sugar dusting because they considered the method too time consuming (35%), impractical (33%) or were worried about the effect of sugar on bees (24%).

Forty-four per cent of participants indicated that they did not want to use drone brood trapping as they considered the method too time consuming (39%) and impractical (20%). Participants were also worried about forgetting to remove drone frames on time (22%), which would lead to accidentally boosting the *Varroa* population in the colony.

### Interviews

In total, we interviewed nine beekeepers, of which seven were commercial, one was part-time and one was a hobbyist beekeeper. Participants kept between 20 and 10 000 colonies. All beekeepers operated in NSW and two had hives located in multiple states.

### Impact and management of *Varroa*

All interviewed beekeepers had *Varroa* in their hives. None reported hive losses due to *Varroa* directly, but rather due to the euthanasia of hives in accordance with the government regulations as part of the early eradication program. One beekeeper downsized his beekeeping business from 1400 to 700 hives to manage *Varroa*.

I just didn't want to have that many bees dealing with Varroa going forward.

With no exception, all interviewed beekeepers used synthetic or organic chemicals to treat *Varroa*. Oxalic acid and Bayvarol (flumethrin) were the methods of choice. Beekeepers indicated trust in chemicals and were mostly satisfied with their use so far.

Once that initial Bayvarol went in … it's done. Trust the chemical, forget about mites, go back to normal for a little while.

However, beekeepers also mentioned negative side effects when using chemicals. Beekeepers observed an increase of queenless hives when treating with chemicals.

Well, I'm not going to say it's Varroa. I'm going to actually say it's the treatments’ part.

Beekeepers noted that although chemical treatments are easy to use, they still demanded significant additional labour and cost. Beekeepers do not get subsidised for the additional cost and labour they must spend to treat *Varroa*.

At the moment we're spending half our beekeeping time controlling Varroa.

Many beekeepers were not satisfied with the proposed management strategies from the government and argued that recommended methods were mainly from countries with different beekeeping conditions and with a natural brood break in winter. A winter brood break occurs in a cold climate where bee colonies stop rearing brood for a few months (Nürnberger et al. 2018). This means *Varroa* cannot hide within capped brood cells and are exposed to chemicals that otherwise do not permeate closed cells, such as oxalic acid (Rademacher and Harz 2006). This situation does not happen in significant beekeeping regions of the country, including the subtropical and tropical parts of Australia.

Beekeepers often perceived neighbouring beekeepers and feral honey bees as major sources of reinfestation following treatment. Trust between beekeepers was rather low—especially when they did not know the other beekeepers present in their surroundings.

[There is] Not a lot of trust in the industry until you get good relationships with trained individuals.

Almost all beekeepers mentioned an increase in secondary pests like small hive beetles (*Aethina tumida*). Beekeepers observed that *Varroa* weakened colonies which made them more susceptible to small hive beetles.

The pest that really combines with Varroa is the small hive beetle, which I'm sure you've heard a lot about. Yes and so,… small hive beetle numbers are higher than they have ever been since I started beekeeping.

### Perception towards biomechanical methods

None of the interviewed beekeepers used biomechanical methods apart from screened bottom boards. These prevent fallen mites from reinfesting the bee colony, unlike solid bottom boards which may allow them to do so (Harbo and Harris 2004). Beekeepers expressed a range of opinions regarding biomechanical methods for managing *Varroa*. While some were open to exploring alternative approaches, others were sceptical about their efficacy, particularly in a commercial context.

I would be happy to try something, but I just can't justify the labour involved in doing it.

Knowledge about non-chemical *Varroa* control methods varied among beekeepers. Some beekeepers considered organic chemicals like oxalic acid as chemical-free methods and knew little about biomechanical controls. Some beekeepers had knowledge of drone trapping, which is presented on a governmental website as a recommended method (Frost 2024). Others were aware of methods, such as heat treatment, sugar dusting and queen caging. Beekeepers often had incorrect information about sugar dusting and believed that bees could drown in the melted sugar, while this issue never appears to be reported by beekeepers practicing sugar dusting.

A high reinfestation rate from the surroundings was another barrier to using biomechanical methods. Beekeepers feared losing colonies, as they were quickly reinfested by *Varroa* after using a biomechanical method. In contrast, chemical treatments often remained in the hive for a number of weeks (e.g., oxalic acid strips), which beekeepers felt was more reliable.

Especially in the phase we're in now—with unstable mite populations—you can enter a coastal area from outside and immediately become heavily infested. If you clean up your bees and take them out of that high-pressure coastal area—say, moving from Forster to Port Macquarie—you’ll likely reintroduce high mite loads and get reinfested. I could treat them [with chemicals] as I exit, and I probably wouldn’t have mites again. So, we’re in this unstable phase. But fast forward five years—mites are everywhere, and there are hot zones with higher mite levels due to beekeeping activity. At that point, you can take your bees out, treat them, and that should be enough. Then, I’d be more interested in looking at non-chemical methods, because the risk of being caught out would be much lower.

A common theme among interviewees was the need for education and demonstration of biomechanical methods. Beekeepers indicated that they would be more motivated to adopt new practices if they were presented with clear, accessible information and real-world examples of success. As one beekeeper put it, *"I need to see it works before I try it myself."*

Additionally, beekeepers did not like the thought of killing brood as necessitated by the brood trapping and queen caging control methods. Beekeepers mentioned that removing brood would deprive the colony of significant resources, making the method very unsustainable.

So, as a beekeeper, I'm a brood producer. I'm paid for my brood and I'm paid for what my brood does, which is either pollinate or collect honey. So, throwing away frames of brood is like saying, I've got this bad leg, I'll just cut it off […]. But I'm now cutting down my production for that.

Some beekeepers reported willingness to consider preventative drift reduction as a control measure, but had concerns about the ease of implementation. Drift reduction can be implemented in various ways, usually spacing hives, painting them with different colours and patterns or changing their orientation (Dynes et al. 2019). Drift reduction is thought to decrease *Varroa* loads in hives and increase winter survival (Dynes et al. 2019). Of these options, beekeepers mentioned using hives painted with different colours but stated that especially for pollination services, spacing hives was impractical due to a lack of space. Seven beekeepers did not consider drifting at all, finding it unimportant.

## Discussion

This is the first study examining beekeepers’ perceptions, trust, and concerns towards biomechanical control methods against *Varroa* after their invasion into Australia in 2022. Our study primarily targeted beekeepers in New South Wales, where the *Varroa* incursion initially occurred, while also including participants from other states to provide broader context for the Australian beekeeping sector.

Beekeepers indicated interest in biomechanical control options; however, they tended to trust and use chemical more than non-chemical options. Although beekeepers noted negative side effects of chemical control options, such as queen failures, chemical control was still their first choice as it remained the cheapest and least time-consuming method to control mites.

The barriers to adopting biomechanical control methods are diverse. Beekeepers reported challenges related to understanding the protocols and time constraints. They also highlighted issues with the practicality of implementing and upscaling methods such as brood removal and comb trapping techniques which take a significant amount of time and must follow a strict calendar regardless of the weather (Büchler et al. 2020). Some beekeepers reported using screened bottom boards as a biomechanical control method and were satisfied with the results. Unlike solid bottom boards, screened boards prevent fallen mites from re-entering the hive, as the mites drop through the mesh onto the ground. However, this method alone is not sufficient for *Varroa* control, as its overall impact on mite populations is relatively low (Jack and Ellis 2021).

A case study from Valentine and Martin (2023), conducted in the UK, gathered 2872 responses, indicated that the majority (72–79%) of UK beekeepers treated hives against *Varroa destructor*. Treatments typically involved two yearly chemical applications. Oxalic acid, thymol, and amitraz were the most popular choices. Only a fraction of beekeepers (3%) reported using biomechanical methods like drone brood removal or sugar dusting as a sole treatment, and only 20% used a combination of chemical and biomechanical treatment methods. While biomechanical methods are often integrated into recommended overall pest management strategies (Rosenkranz et al. 2010; Whitehead 2017; Jack and Ellis 2021), the level of adoption of these methods among commercial beekeepers remains unclear worldwide and is low in Australia. Studies on the use of biomechanical methods are scarce (O’Connell et al. 2025), which limited our ability to compare perceptions of these methods between Australia and other countries.

Comparative studies, such as those by Thoms et al. (2019) and Kahane et al. (2022), who studied the motivation, philosophy and beliefs of beekeepers towards *Varroa* management revealed differences among beekeepers, particularly in the USA and the UK. Thoms et al. (2019) identified two primary categories: "Treatment Skeptics", who prefer minimal intervention and avoid chemical treatments and "Treatment Adherents", who actively manage their hives and are more likely to use chemical treatments. While all “Treatment Skeptics” were hobbyist beekeepers, “Treatment Adherents” were a mix of commercial, part-time and hobbyist beekeepers. This trend is similar in Kahane et al. (2022) findings, which suggests that commercial beekeepers are more motivated to use chemical treatments, while certain hobbyist groups actively avoid them. Underwood et al. (2019) suggested a similar trend in the USA, indicating that commercial and sideline beekeepers utilized more chemicals than hobbyists, who often explored a broader range of treatment options, including organic acids and essential oils.

Contrary to these studies, our survey did not reveal significant differences in *Varroa* management practices among commercial, semi-commercial, and hobbyist beekeepers. This may be attributable to the very recent arrival and spread of *Varroa* in Australia. Beekeepers perceive the chemical control methods as conventional and likely to yield better, more consistent results against *Varroa*. These methods may also appear simpler or less technical, and appeal to users with limited knowledge of alternative control options prior to the invasion. Moreover, the low awareness of prevention methods such as drift management among beekeepers is concerning. Only two of the nine interviewed beekeepers considered drift when placing their hives, despite evidence suggesting that crowded apiaries increase vulnerability to *Varroa* infestations (Seeley and Smith 2015; Dynes et al. 2019). This indicates either a lack of knowledge regarding prevention methods or a perception that their importance is minimal, which could exacerbate the challenges posed by *Varroa*.

Some beekeepers might also incorrectly believe they already had other biomechanical control options in place. In our interviews, beekeepers often regarded organic methods like oxalic acid as non-chemical options, showing a need for clearer, more available information about chemical treatments. Interviewed beekeepers stated they were unsatisfied with the available learning options presented by the government, missing clear information sources and guidance towards *Varroa* management. Government websites and workshops are the main information source of information for *Varroa* treatment and were so far mainly based on information obtained from New Zealand, the USA, and Europe. However, beekeepers expressed a desire to obtain information derived from Australian data and specifically targeting Australian conditions.

De Carolis et al. (2023) conducted an international online survey designed as a risk assessment tool to understand beekeepers' knowledge and adoption of good beekeeping practices and biosecurity measures for *Varroa* management. In this study, the authors state that 80% of their participants in Europe and the Americas treated their hives with chemicals (20% did not treat for *Varroa*). The survey revealed a high interest (89.9% in the Americas, 82.8% in Europe) among beekeepers for additional bee health training and a willingness to connect with veterinary experts specialised in bees. While De Carolis et al. (2023) did not include biomechanical control methods in their survey, the study demonstrates a widespread use of chemical treatments in these regions.

In South America, countries like Argentina and Chile rely on intensive chemical treatments which often results in miticide-resistant mites and colony loss (Maggi et al. 2016). However, in tropical and subtropical regions, like Venezuela and Brazil, the presence of Africanized honeybees has improved colony survival, as their hygienic and grooming behaviours provide a more natural resistance compared to European honey bees. In these regions, the mite is less virulent, often allowing coexistence without chemical intervention (Maggi et al. 2016). Although African and Africanised honey bees are known to be *Varroa*-resistant, they are often rejected by beekeepers in the Northern Hemisphere due to their more defensive and aggressive behaviour, as well as their tendency to swarm and abscond frequently, which makes them less desirable for commercial beekeeping (Guzman-Novoa et al. 2024).

In Australia, all interviewees reported using chemicals as their first choice. To address these issues, developing practical biomechanical control methods for both hobbyist and commercial beekeepers is essential. Providing accessible, easy-to-understand protocols will facilitate the adoption of these methods. Structural drivers of chemical reliance include limited access to locally relevant information, time constraints, and reliance on conventional practices recommended by extension services. Our study showed that most beekeepers gathered information about *Varroa* treatments via beekeeper clubs, associations, and government sources, which should be considered when providing new information. Similarly, outreach activities should be more targeted, as proposed by Thoms et al. (2019), and should take into account beekeepers’ beliefs (e.g., commercial beekeepers are more likely to rely on chemical treatments). Hands-on demonstration events and field trials are recommended, as they provide practical experience and build trust, addressing the barrier of low familiarity with non-chemical methods. Verbeke et al. (2024) have shown that the roles of networks and peers are important when adopting new technology in the beekeeping sector. Working with beekeepers who showed willingness to implement and adopt new methods is key, as they may act as exemplars for other beekeepers in their communities.

Our study relied on self-reported information from beekeepers, which may be subject to recall and social desirability bias. Nevertheless, such surveys are widely used in apicultural research and provide essential insights into management practices that would otherwise be difficult to capture at large scales. We aimed to identify general associations rather than precise estimates, which helps to mitigate the potential impact of these biases on our conclusions.

## Conclusions

The arrival of *Varroa destructor* in Australia marks a major change for the beekeeping industry. While chemical treatments are still widely used, many beekeepers are already showing interest in non-chemical management options. To support this change, all the barriers faced by beekeepers must be addressed. In Australia, we have identified these barriers to be a lack of information, time, or trust in alternatives. Education, peer-to-peer exchange, and practical resources in the form of information sheets about biomechanical methods could help make these options more accessible. As the Australian beekeeping industry adapts to this new challenge, building trust, developing practical methods, sharing knowledge, and reducing boundaries between research, policy, and practice will be key to finding solutions that are both effective and sustainable in the short and long term.

## Supplementary Information

Below is the link to the electronic supplementary material.

Supplementary file1 (PDF 684 KB)

## Data Availability

Research data supporting this publication are available as supplementary information (Appendix S1). Due to ethical reasons, interview transcripts cannot be made openly available.
