# Supplementary material for: Beekeeper attitudes towards the management of Varroa destructor in Australia
Source: Ambio. 2025 Dec 11;55(6):1293–303. doi: 10.1007/s13280-025-02307-5 (PMC13125557; doi:10.1007/s13280-025-02307-5)
Supplement: Supplementary file 1 — Supplementary file1 (PDF 684 KB) [file 13280_2025_2307_MOESM1_ESM.pdf]

# Supplementary Information

## Beekeeper attitudes towards the management of Varroa destructor in Australia

Cornelia Sattler[1*], Andrew B. Barron[1], Theotime Colin[1]

[1]School of Natural Sciences, Macquarie University, New South Wales, Australia

*Corresponding author: cornelia.sattler@mq.edu.au

**S1**: Online survey

**S2**: Semi-structured interviews

**S1: Online survey**

# Innovative methods for the management of Varroa destructor in Australia

Do you consent to take this survey?

- o Yes
- o No

I agree to the future use of data for extended consent to use my deidentified data for future research projects that are similar to this project and have Human Research Ethics Committee approval.

- o Yes
- o No

End of Block: Introduction

Start of Block: Role classification

**Where is your main location of your largest apiary? Please provide a postcode.**

______

End of Block: Role Classification

Start of Block: Level of experience

**1. Level of expertise**

**B.Q. 1.0 Which of the following best describes your beekeeping operation?**

- o Hobbyist or amateur
- o Semi-commercial, part-time beekeeper
- o Full-time commercial beekeeper

**B.Q.1.1 What is the main service you are focusing on? What percentage of your business is:**

- o Pollination
- o Honey production
- o Queen rearing or colony propagation
- o Others:________

**B.Q.1.2 How many colonies do you have? Please enter numbers only.**

- o Full hives
- o Nucleus
- o Queen rearing hives

**B.Q.1.3 Do you harvest honey?**

- All year around
- During spring and summer
- Other:

End of Block: Level of expertise

---

Start of Block: Varroa methods

**2. Varroa monitoring and methods**

**B.Q.2.1 How do you gather information about varroa treatment? (select all that apply)**

- Via a beekeeping mentor
- Via a club or association
- Via government communications or workshops (i.e. DPI)
- Via a beekeeping shop
- On beekeeping websites
- On social media (i.e. Facebook groups)
- On YouTube or other video streaming platforms
- Via podcasts
- Others: ________________________

**B.Q.2.3 How often do you check for varroas in your hives?**

- More than once a month
- Once a month
- Once every three months
- Once every six months
- Once a year
- I don't check for mites
- Others:

**B.Q.2.4 How many colonies did you lose due to varroa mites in the last year (excluding loss due to eradication strategy)? Please enter numbers only.**

Estimate: ___________

**B.Q.2.7 Did the following varroa mite treatment meet your satisfaction?**

| | Yes | No | Did not use |
|---|---|---|---|
| Apivar/Apitraz (Active ingredient: Amitraz) | | | |

| |
|---|
| Apistan (Active ingredient: Fluvalinate) |
| Bayvarol (Active ingredient: Flumethrin) |
| Formic Pro (Active ingredient: Formic acid) |
| Apiguard (Active ingredient: Thymol essential oil) |
| Dusting (powdered sugar) |
| Api Bioxal (Active ingredient: Oxalic acid) |
| Oregano oil |
| Eucalypt oil |
| Menthol |
| Rhubarb leaves |
| Artificial swarming |
| Brood breaks via queen caging |
| Brood breaks via cold storage |
| Dusting of powdered sugar |
| Others |

**3. Rating of alternative methods**

**B.Q.3.1 In the following section we would like to get your feedback on non-chemical (alternative) methods against varroas. Please rate how strong you agree or disagree with the following statements.**

| | Strongly disagree | Disagree | Neither agree or disagree | Agree | Strongly agree |
|---|---|---|---|---|---|
| I have not heard of alternatives to chemical control methods before taking this survey | | | | | |
| I have tried non-chemical control methods and they did not work for me | | | | | |
| I do not trust alternative to chemical treatments | | | | | |
| Alternative methods take too much time | | | | | |
| The protocols of alternative methods are difficult to find | | | | | |
| The protocols of alternative methods are difficult to understand | | | | | |
| I would be open to alternative methods if somebody can teach me or demonstrate the method | | | | | |
| Alternative methods are bad for the bees | | | | | |
| Alternative methods contaminate honey | | | | | |

**B.Q.3.2 We will now present three example protocols for chemical free control methods of varroa mites. With these examples we want to understand what barriers you would see to adopting these methods if they are proven to be effective against varroas.**

**Please note that the three presented protocols are examples only which we do not intent to recommend at this initial trial stage.**

Method 1: Trapping Comb Method

The aim of this method is to capture all Varroa mites in worker brood, remove them, and freeze the frames. To achieve this, the queen is caged on a single frame three times consecutively so that she can only lay eggs on designated frames. These frames are then removed and frozen. This operation lasts 27 days to cover a full brood cycle. The general idea is to ensure that only these three frames have open cells and that all the Varroa mites are successfully trapped in these frames.

Here is a proposed timeline for this methodology:

Day 1: The queen is caged on a single frame inside the brood box.

Day 9: The queen is moved and caged on a second frame inside the brood box.

Day 18: The queen is moved and caged on a third frame inside the brood box. The brood on the first frame is now fully capped. The frame is removed and frozen for 48 hours to kill the mites, then returned to the beehive.

Day 27: The queen is freed. The second frame is frozen for 48 hours to kill the mites and returned to the beehive.

Day 35: The third frame is removed and frozen for 48 hours before being returned to the hive. This marks the end of the trapping method.

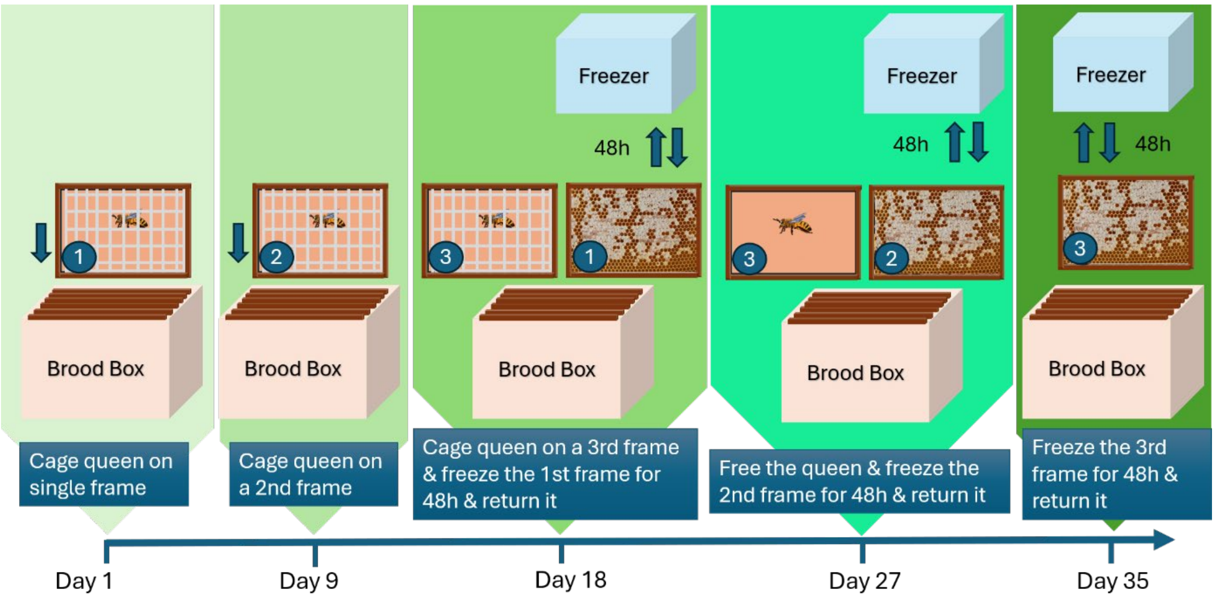


If this method is working, would you use it over chemical control?

- Yes
- No

If not, why wouldn't you use it? (select all that apply)

- this method is hard to understand
- this method is unpractical
- This method is time-consuming
- I do not want to kill bee larvae
- I am worried about the queen's survival
- Other:

Method 2: Icing sugar dusting. In this technique, 15g of powdered icing sugar is blown using an air blower in between the frames of the brood box, without separating the frames, every three days for a month (9 treatments). Icing sugar is believed to dislodge varroa mites or cause a grooming in bees which then dislodge mites.

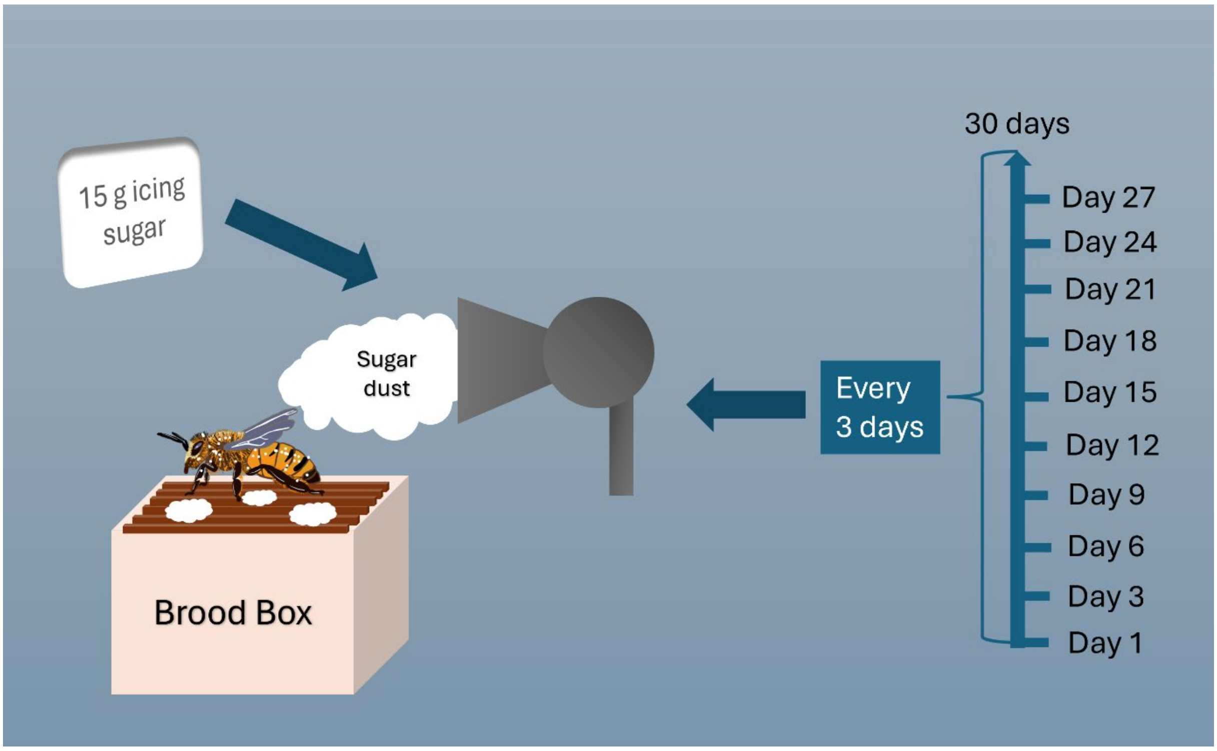


If this method is working, would you use it over chemical control?

- Yes
- No

If not, why wouldn't you use it? (select all that apply)

- this method is hard to understand
- this method is unpractical
- This method is time-consuming
- I am worried for the health of the colony if I were to use this method

- I am worried about the effect of icing sugar on bees
- I am worried about the queen's survival
- I am worried about honey contamination with icing sugar
- Other:

Method 3: Trapping of varroa mites in drone cells. In this technique, drone foundation is introduced into colonies to encourage drone egg laying and the foundations are removed before drones' hatch, between 12 and 22 days after the drone frames were introduced in hives. Drone cells are believed to be more attractive to varroa mites, and removing and freezing frames of capped drone brood may reduce the number of mites in hives.

In total, 4 trap frames are introduced in hives:

Day 1: trap frame 1 is introduced

Day 7: trap frame 2 is introduced

Day 14 trap frame 3 is introduced, trap frame 1 is removed

Day 21 trap frame 4 is introduced, trap frame 2 is removed

Day 28 trap frame 3 is removed

Day 35 trap frame 4 is removed

Removed drone frames are either uncapped with a fork and drone pupae removed and destroyed, or the whole frames are frozen for 48h, thawed and reintroduced in hives for bees to clean for another 48h before storing them in a dry place.

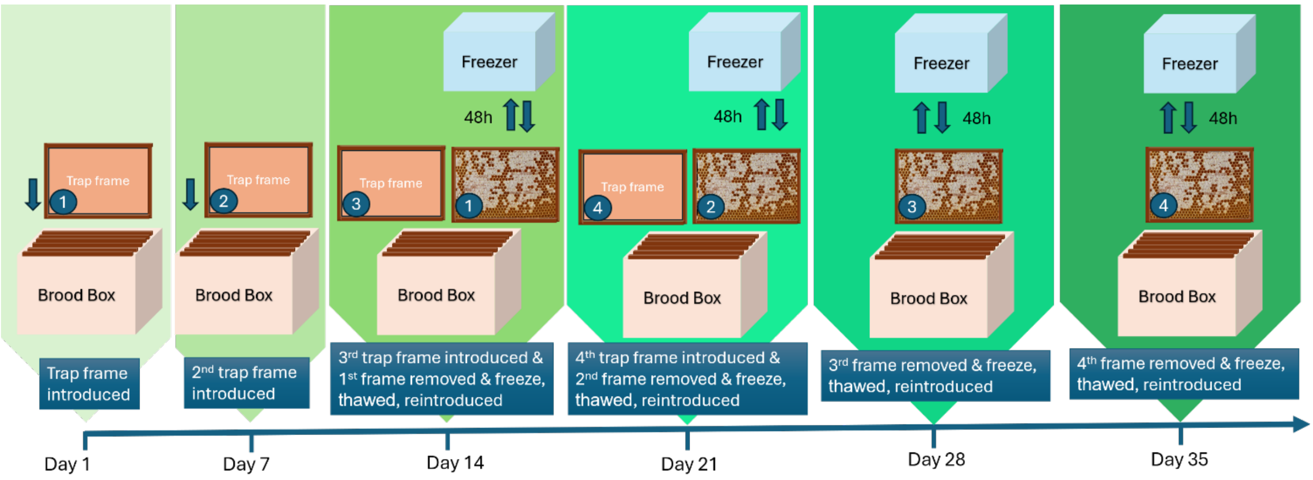


If this method is working, would you use it over chemical control?

- Yes
- No

If not, why wouldn't you use it? (select all that apply)

- this method is hard to understand
- this method is unpractical
- This method is time-consuming
- I am worried for the health of the colony if I were to use this method

- I am worried about the management and storage of frames
- I am worried about forgetting or not being able to remove drone frames on time
- I do not want to kill drone brood
- Other:

End of Block: Varroa

---

**B.Q.3 We appreciate the time you've taken to complete this online survey. If you have any comments or thoughts, you'd like to share with us, please use the text field below.**

**S2: Semi-structured interview questions**

# Innovative methods for the management of *Varroa destructor* in Australia

Can you tell me about your background and where you are located?

1. Do you currently have varroa mites in your hives?
2. How often do you check varroa levels?
3. How do you obtain information about varroa control?
    1. How could this be improved?
4. Did you experience absconding bees or notice any pests that you think might increase due to Varroa? (For example, a combined effect making the situation worse.)
5. What methods do you currently use to treat varroas?
6. How satisfied are you with the methods you're using right now? Do you feel that they are efficient? How do you assess effectiveness?
7. Would you consider trialling chemical-free methods?
8. Do you know chemical-free methods?
    1. Which chemical free methods are you most interested in, if any? Which ones do you see as unlikely to work?
9. What are the main barriers to adoption of chemical free varroa control?
10. What would be an acceptable varroa control method, in terms of costs, time, efficacy?
11. Do you consider drifting in your varroa management?
12. Is there any topic or aspect you expected us to cover that we haven’t addressed yet?

Can you provide three potential contacts for further interviews?
